# Eicosapentaenoic Acid as long-term secondary prevention after ischemic stroke

**DOI:** 10.1186/s40169-015-0062-5

**Published:** 2015-06-11

**Authors:** Taizen Nakase, Masahiro Sasaki, Akifumi Suzuki

**Affiliations:** Department of Stroke Science, Research Institute for Brain and Blood Vessels –Akita, 6-10 Sensyu Kubota Machi, Akita, 010-0874 Japan

**Keywords:** Stroke recurrence, Secondary prevention, Anti-platelet agent, Eicosapentaenoic acid

## Abstract

**Background:**

It is sometimes difficult to choose anti-thrombotic agents for secondary prevention in stroke patients at high bleeding risk. Recently, Eicosapentaenoic Acid (EPA) was reported to reduce the recurrence of stroke in hypercholesterolemic patients without increasing hemorrhagic risk. In this study, we investigated the features of recurrent stroke patients during EPA medication as secondary stroke prevention.

**Methods:**

Following the approval of the ethical committee, stroke patients in the outpatient clinic were consecutively screened and patients who continuously take EPA were enrolled in this study (n = 71, average age 69.7 yo). Blood sample data was adopted from the latest visit or the admission at the stroke recurrence. According to the previous stroke history, all patients were classified into the hemorrhagic stroke (HS) group (n = 10) and the ischemic stroke, including asymptomatic infarction, (IS) group (n = 61).

**Result:**

Any stroke recurrence was not observed in the HS group. Whereas, ischemic stroke recurrence was observed in 6 patients in the IS group, although there was no hemorrhagic stroke recurrence. Recurrent stroke patients showed the higher serum level of cholesterol or the renal dysfunction. The stroke subtype of patients were 2 embolic strokes, 3 atherothrombotic infarctions (two were compromised with renal failure and one had insufficient amount of EPA) and one lacunar infarction (who showed high triglyceride level).

**Conclusion:**

Hemorrhagic stroke was not occurred in our observation of EPA prescribed patients. The clinical features of recurrent stroke patients were the existing complications of dyslipidemia and renal dysfunction.

## Background

Eicosapentaenoic Acid (EPA) is one of essential fatty acid and is contained mainly in fish oil. EPA has been reported to not only reduce serum level of triglycerides and low-density lipoprotein [1, 2], but also inhibit platelet aggregation [3, 4] and reduce the arterial wall thickening [5]. In the results of Japan EPA Lipid Intervention Study (JELIS) sub-analysis, EPA could decrease the rate of stroke recurrence without increasing the risk of hemorrhagic incidence [6].

Meanwhile, as the medication for secondary stroke prevention, anti-platelet agents are commonly prescribed [7]. However, some patients might suffer from recurrence of ischemic stroke. Or, sometimes, intracerebral hemorrhage (ICH) might occur on the ischemic stroke survivors who are taking anti-thrombotics in higher rate compared with patients without any anti-thrombotics [8]. Even, hypertensive ICH patients sometimes have to take anti-thrombotic agents, because they may have arteriosclerotic lesions. Therefore, it will become difficult to choose anti-thrombotic agents for the patients, such as ischemic stroke patients with high hemorrhagic risks and ICH patients with severe arteriosclerotic lesions. Of course, atherosclerotic risks are concurrently treated with anti-thrombotic medications, and some medicines of risk factor treatment, such as antihypertensive drugs and lipid-lowering drugs, have been reported to show the effect of stroke prevention [9].

In this context, EPA could be a candidate for the additional anti-thrombotic agent. Since JELIS was a large-scale cohort study, it is unclear that which type of patients was appropriate or not appropriate for the use of EPA as preventing stroke recurrence. Thus far, for being a help of discussing the optimal medication with EPA, we revealed the clinical features of patients who were suffered from stroke recurrence under EPA prescription.

## Methods

### Patients

Following the approval of the ethical committee of Research Institute for Brain and Blood Vessels –Akita (the document number is #14-20), stroke patients in the outpatient clinic were consecutively screened during a month of October 2013. Patients with a history of stroke or patients with diagnosing asymptomatic brain infarction and continuously prescribed EPA for more than one month were enrolled in this study. Past history and medications were collected from clinical records. Vascular risk factors were defined as follows: hypertension (>140 mmHg of the systolic BP or >90 mmHg of the diastolic BP, or currently prescribed anti-hypertensive drugs), diabetes mellitus (casual blood glucose level >200 mg/dL or currently prescribed anti-diabetic medication), hyperlipidemia (>220 mg/dL serum total cholesterol or >150 mg/dL triglyceride, or currently prescribed anti-dyslipidemic medication), alcohol drinking (>30 ml of alcohol conversion amount per day) and smoking. Renal function was assessed by calculating the estimated glomerular filtration rate (eGFR), using the Japanese Society of Nephrology formula [10]. Duration of EPA medication was calculated in each patient. Stroke recurrence was set as the end point. Blood sample data was adopted from the laboratory examination of the latest visit or the admission at the stroke recurrence. According to the previous stroke history, all patients were classified into the hemorrhagic stroke (HS) group (n = 10: patients with past ICH history) and the ischemic stroke (IS) group (n = 61: patients with brain infarction history, including asymptomatic infarction).

### Statistical analysis

Data are presented as mean ± standard deviation (SD) for continuous variables and percentage (%) for categorical variables. To extract the features of recurrent stroke patients, clinical backgrounds were compared between asymptomatic patients, brain infarction (BI) with no recurrent patients and BI with recurrent patients in the IS group by the non-parametric one-way ANOVA test for continuous variables and by the Pearson χ^2^ test for categorical variables. All statistical analysis was performed by JMP9 software (SAS Institute Inc., Cary, NC).

## Results

As a total, 71 patients were enrolled in this study (male 44, female 27, average age: 69.7 ± 12.1 yo). The duration of taking EPA was 14 years as maximum and 1 month as minimum (average: 4.19 years). Clinical backgrounds of all patients in the HS group were shown in Table 1. There was no stroke recurrence in this group. Because the number in each group was small, statistical analysis was not applied. Clinical backgrounds of all patients in the IS group was shown in Table 2. Although none of hemorrhagic stroke was occurred, 6 ischemic stroke recurrences were observed. These all recurrent cases were not from the asymptomatic infarction but from the BI patients. Then, the clinical characteristics were compared between asymptomatic patients, no recurrent patients (BI-no recurrence) and recurrent patients (BI-recurrence). The level of serum triglyceride was significantly higher in the BI-recurrence compared with other groups (p = 0.046). The amount of serum creatinine was significantly higher in the BI-recurrence compared with other groups (p = 0.001). There was a significant decline of eGFR in the BI-recurrence compared with other groups (p = 0.008). Combination use of anti-thrombotic agents was significantly in low frequency in asymptomatic patients (p = 0.001).

**Table 1:** Clinical backgrounds of the HS group

	ICH	ICH + BI	BI + ICH
N	6	2	2
Sex (F/M)	1/5	0/2	2/0
Age	63.5 ± 10.4	78.5 ± 9.2	71.0 ± 7.1
EPA (mg)	1950 ± 677	2288 ± 636	2372 ± 636
Length (years)	2.4 ± 1.0	5.1 ± 1.8	2.6 ± 0.6
Hypertension (%)	100	100	100
Hyperlipidemia (%)	83.3	50	100
DM (%)	16.7	0	100
Af (%)	16.7	0	0
TCho (mg/dl)	198.2 ± 21.5	157.0 ± 22.6	186.5 ± 57.3
LDL (mg/dl)	120.2 ± 18.6	87.0 ± 7.1	117.5 ± 57.3
TG (mg/dl)	89.5 ± 33.7	76.0 ± 18.4	100.0 ± 4.2
Cre (mg/dl)	0.7 ± 0.1	0.8 ± 0.1	0.7 ± 0.1
eGFR (ml/min/1.73 m^2^)	79.6 ± 11.9	76.2 ± 5.5	64.4 ± 16.1
AP, single (%)	0	0	50
AP, multi (%)	16.7	0	0
Warfarin (%)	16.7	0	0
W + AP (%)	0	50	0
None (%)	66.7	50	50

*ICH* intracranial hemorrhage, *BI* brain infarction, *DM* diabetes mellitus, *Af* atrial fibrillation, *T Cho* total cholesterol, *LDL* low-density lipoprotein, *TG* triglyceride, *Cre* creatinine, *eGFR* estimated Glomerular Filtration Rate, *AP* anti-platelet, *W* warfarin

**Table 2:** Clinical backgrounds of the IS group

	Asymptomatic	BI-no recurrence	BI-recurrence	p value
N	13	42	6	
Sex (F/M)	8/5	13/29	3/3	0.122
Age	72.1 ± 11.7	68.4 ± 12.7	76.5 ± 9.9	0.262
EPA (mg)	2077 ± 432	1907 ± 407	1650 ± 677	0.151
Length (years)	4.7 ± 4.2	3.9 ± 4.2	7.3 ± 5.4	0.200
Hypertension (%)	76.9	71.4	66.7	0.883
Hyperlipidemia (%)	83.3	85.0	100	0.500
DM (%)	75.0	42.9	33.3	0.191
Af (%)	7.7	9.5	16.6	0.824
TCho (mg/dl)	190.7 ± 46.8	170.3 ± 32.5	201.8 ± 28.2	0.054
LDL (mg/dl)	114.7 ± 39.7	96.9 ± 27.0	120.5 ± 43.2	0.088
TG (mg/dl)	140.8 ± 69.3	119.6 ± 53.5	248.5 ± 325.9	0.046
Cre (mg/dl)	0.7 ± 0.2	0.7 ± 0.2	1.1 ± 0.5	0.001
eGFR (ml/min/1.73 m^2^)	74.5 ± 18.8	85.0 ± 21.9	52.7 ± 23.3	0.008
AP, single (%)	7.7	45.2	50.0	0.041
AP, multi (%)	0	19.0	16.7	0.096
Warfarin (%)	7.7	7.1	0	0.536
W + AP (%)	0	4.8	0	0.490
None (%)	84.6	21.4	16.7	0.001

*BI* brain infarction, *TCho* total cholesterol, *LDL* low-density lipoprotein, *TG* triglyceride, *Cre* creatinine, *eGFR* estimated Glomerular Filtration Rate, *AP* anti-platelet, *W* warfarin

Because the number of stroke recurrent patients was small and the power of statistical analysis was weak, we precisely observed all recurrent patients for revealing the remarkable features in these patients. Two patients showed the cardioembolic stroke. A 78 years-old woman was prescribed EPA and a beta1 receptor blocker (metoprolol), due to the asymptomatic brain infarction observed by a health examination in 1 year and 7 months ago. She had no atrial fibrillation history but had hypertension, hyperlipidemia and diabetes mellitus as stroke risks. Another woman was 70 years-old and was prescribed EPA and an angiotensin II receptor blocker (ARB: candesartan) for 2 years and 9 months. She had atrial fibrillation and hyperlipidemia as stroke risks. She had previous brain infarction 4 months ago. Other three patients were the atherothrombotic infarction. An 85 years-old man had atherothrombotic infarction 3 years ago and was prescribed EPA and an anti-platelet agent (clopidogrel). He had hypertension and hyperlipidemia as stroke risks and showed the increased amount of creatinine (1.4 mg/dl). A 91 years-old man had a history of lacunar infarction and was prescribed EPA, aspirin, clopidogrel, calcium channel blockers (CaB: benidipine and nifedipine) and a dipeptidyl peptidase-4 inhibitor (sitagliptin) for 4 years and 10 months. He had hypertension, hyperlipidemia and diabetes mellitus as stroke risks. Moreover, he showed the increased amount of creatinine (1.8 mg/dl). A 69 years-old woman had hyperlipidemia and a history of cardioembolic infarction and was prescribed EPA for 5 years and 9 months. However, the prescribed dose of EPA (900 mg per day) was lower than the recommended standard amount (1800 mg per day). The last one patient was 66 years-old man. His stroke subtype was the lacunar infarction. He had hypertension, hyperlipidemia and diabetes mellitus as stroke risks. He had a history of lacunar infarction two years ago, and was started the prescription of EPA, an anti-platelet agent (cilostazol), rosuvastatin, an ARB (valsartan) and a CaB (amlodipine). Although he had taken EPA and statin, his triglyceride amount was extremely high (911 mg/dl).

## Discussion

This study clearly indicated the clinical features of the recurrent ischemic stroke patients who were treated with EPA as secondary stroke prevention. The recurrent ischemic strokes had been accompanied by the complications of dyslipidemia or renal dysfunction, in spite of EPA medication.

Actually, EPA is one of essential fatty acids and can be purchased as a dietary supplement. However, the extra purified EPA was developed as an anti-hyperlipidemia agent and can be prescribed as an officially approved medicine in Japan [11]. This concentrated EPA would be suitable for assessing the medical effects in the clinical situation. According to the observational study, Greenlanders who had n-3 polyunsaturated fat rich diet showed a reduced level of serum cholesterol and triglyceride, resulting in a low incidence of acute myocardial infarction [12, 13]. On the contrary, meta-analysis of cohort studies revealed that there was no consistent evidence of the reduction of cardiovascular events by the omega-3 fats intake [14]. Meanwhile, JELIS trial had showed that the incidence of major cardiovascular events was significantly reduced in the hyperlipidemia patients who treated with EPA addition to statin compared with the hyperlipidemia patients who treated with only statin [15]. In clinical situation, it was reported that the serum level of adiponectin was significantly increased by EPA administration in type 2 diabetic patients [16]. Metabolic syndrome patients treated with EPA showed a significant reduction of small dense LDL and C-reactive protein compared with those with diet alone [2]. JELIS sub-analysis indicated that EPA prescription was more effective for preventing the stroke recurrence than for primary prevention of stroke. Moreover, there was no increase of hemorrhagic incidence in the EPA treated group compared with the control group [6]. Usually, anti-platelet agents are prescribed for the prevention of non cardioembolic stroke [7]. Even though, if a patient taking aspirin or clopidogrel suffered from brain infarction as ischemic stroke recurrence, it might be puzzling whether continuing current prescription or changing to different medicine. In that situation, the additional prescription of EPA can be expected to reduce the risk of stroke recurrence without increasing the risk of hemorrhagic complications. Therefore, to be a help of discussing the optimal medication with EPA as secondary stroke prevention, we precisely described the features of each recurrent stroke patients.

Originally, EPA has been reported to reduce the amount of LDL and triglyceride [1, 2]. Moreover, the intake of EPA could increase the EPA/arachidonic acid (AA) ratio, leading to the positive regulation of prostaglandin (PGI) and thromboxane (TXA) [4]. The increase of EPA was reported to decrease the amount of AA in platelet and reduce the TXA2 dependent platelet aggregation [17]. According to our findings, recurrent patients had significantly higher serum triglyceride level compared with no recurrent patients. One patient showed extremely high triglyceride level in spite of taking EPA. Moreover, patients who had stroke recurrence showed the increased amount of serum creatinine and the decreased eGFR. The impaired renal function, that is, chronic kidney disease (CKD) could be a risk of stroke [18, 19]. Meanwhile, EPA could reduce the oxidation of LDL and the lipid hydroperoxide level in vitro, suggesting an anti-inflammatory effect of EPA [20]. Therefore, the effect of EPA in patients with CKD will be investigated in the future study. Furthermore, there is a possibility of EPA for reducing the risk of atrial fibrillation by enhancing the vascular membrane stability. This effect might be good for preventing embolic stroke indirectory. However, we observed two embolic brain infarction as recurrent stroke. It is another assignment to reduce the risk of embolic stroke as stroke recurrence in patients with atrial fibrillation.

Actually, because all data was retrospectively collected from patient’s record, the prescribed EPA dose was not standardized. Moreover, the duration of EPA medication was diverted, i.e., from 1 month to 14 years. Then, the recurrent cases were treated with EPA for from 4 month to 5 years. When exploring the effect of EPA for reducing stroke recurrence, the duration of treatment should be considered. In the JELIS trial, the difference of the accumulated recurrence rate between EPA treated and non-EPA treated groups became prominent after 2 years. Therefore, this study could not mention the results of the effectiveness of EPA as secondary stroke prevention.

This study was a retrospective and the subjects were only the patients who had continuously prescribed EPA agent in our clinic. Therefore, there is a possibility of overlooking the patients who had discontinued EPA prescription after stroke recurrence or who had been lost the follow up. Moreover, all data of the patients were collected from outpatient clinic. It means that there is a possibility in which patients might not take EPA as the same as our prescription. It would be a bias of influencing on the effect of EPA. However, this situation could be experienced in usual practice. Furthermore, since EPA can be contained in various foods, especially fish oil, the serum EPA level may be influenced by daily food intake. Considering the patients in our study, all are from neighboring community and their generation is close. That means they are assumed to have similar daily foods. Therefore, although we had not measured fatty acid fractionation in this study, it can be considered that our data might not be affected by patient’s diet. Even though taking these limitations into consideration, our data could detect the clinical features of recurrent stroke patients.

## Conclusion

ICH as stroke recurrence was not observed in the patients with EPA medication. In our observation, the recurrent ischemic stroke patients who had been taking EPA showed the complications of dyslipidemia and renal dysfunction.
